# Magnolol Inhibits the Growth of *Cryptococcus neoformans* by Disrupting the GSH Oxidation–Reduction System

**DOI:** 10.1155/cjid/8258892

**Published:** 2025-10-05

**Authors:** Tiantian Wang, Pingchuan Li, Fenghua Zhang

**Affiliations:** ^1^Center for Clinical Laboratory, The Affiliated Taian City Central Hospital of Qingdao University, Taian 271000, Shandong, China; ^2^Department of Laboratory Medicine, Zhoupu Hospital Affiliated to Shanghai University of Medicine and Health, Shanghai 201318, China

**Keywords:** *Cryptococcus neoformans*, magnolol, metabolomics, transcriptomics

## Abstract

**Objective:**

In this study, *Cryptococcus neoformans* BNCC225501 was used as research objects to explore the antifungal activity of magnolol. The molecular mechanisms underlying magnolol against *Cryptococcus neoformans* BNCC225501 were explored using metabolomics and transcriptomics. The results offer ideas and directions for developing new antifungal drugs based on magnolol.

**Method:**

The minimum inhibitory concentration (MIC) and the method of drawing the growth curve were used to determine whether magnolol had fungistatic and fungicidal effects on *Cryptococcus neoformans*. *Cryptococcus neoformans* was cultured on urea basal medium and Niger seed medium, and different concentrations of magnolol were added to these media to detect the effects of magnolol on the urease and melanin of *Cryptococcus neoformans*. Network pharmacology was used to screen for potential target genes of magnolol and fungal infection, obtaining common targets. Further molecular docking was performed between magnolol and the proteins corresponding to the common targets.

**Results:**

The MIC of the individual tested agents against *Cryptococcus neoformans* strain was 8 μg/mL for magnolol. The combination of magnolol and fluconazole revealed good synergistic effects against *Cryptococcus neoformans* (FIC ≤ 0.5). At a magnolol concentration of 8 μg/mL, the expression of urease was inhibited. Compared to the control group, in the experimental group treated with magnolol, among the differential metabolites, lipids and lipid-like molecules accounted for the highest percentage of differential metabolites, except for unclassified metabolites. A total of 30% of the metabolites belonged to lipids and lipid-like molecules, followed by amino acids, peptides, and analogues (22.63%), organoheterocyclic compounds (7.91%), alkaloids (6.17%), sugars (6.01%), nucleosides, nucleotides and analogues (3.80%), organic oxygen compounds (2.22%), aromatic compounds (1.90%), organic acids (1.90%), and other categories (16.93%). KEGG pathway enrichment analysis results showed that the metabolites were mainly enriched in glyoxylate and dicarboxylate metabolism, arginine biosynthesis, valine, leucine, and isoleucine degradation; arginine and proline metabolism; cysteine and methionine metabolism; pentose and glucuronate interconversions; sphingolipid metabolism; histidine metabolism; and the citrate cycle. Compared to the control group, in the experimental group treated with magnolol, a total of 928 genes were detected to be altered. The GO enrichment results showed that the altered genes were enriched in the component of membrane and oxidation-reduction process. KEGG enrichment analysis showed that the differential genes were mainly enriched in the DNA replication, NOD-like receptor signaling pathway, and fatty acid biosynthesis. Combined analysis of metabolomics and transcriptomics demonstrated that the glycerophospholipid metabolism pathway and glutathione metabolism pathway were significantly changed after treatment with magnolol. Screening identified 31 target genes for magnolol and 969 fungal genes, with three intersecting targets, DPP4, MAPK14, and CCNA2. Molecular docking studies showed that magnolol and the target protein had a good binding ability, a docking binding energy of −7.3 kcal/mol.

**Conclusions:**

Magnolol has fungistatic and fungicidal ability for *Cryptococcus neoformans* and can inhibit the urease production capacity of *Cryptococcus neoformans*. The antifungal mechanism of magnolol may be that it disrupts redox homeostasis and inhibits detoxification by affecting Glutathione metabolism.

## 1. Introduction

*Cryptococcus neoformans* is widely distributed in nature, and there are more than 30 species in the genus *Cryptococcus*, of which only *Cryptococcus neoformans* and *Cryptococcus gattii* are associated with human diseases [1]. In China, *Cryptococcus* infections are predominantly *Cryptococcus neoformans* [2]. Soil is an important place for *Cryptococcus* to host, and *Cryptococcus* is often found in bird feces, especially pigeon feces.

*Cryptococcus* can invade various organs of the body, most commonly the nervous system, followed by the lungs and skin, and can cause cryptococcal meningitis and pulmonary cryptococcosis. Because fungal infections often require long-term treatment, and the long-term use of antifungal drugs commonly used in the clinic has obvious drawbacks, such as the tendency to produce drug-resistant strains and serious toxic side effects on host cells [3].

These have greatly increased the difficulty of treating *Cryptococcus neoformans* infections, and the development of new effective therapeutic agents is very urgent. Magnolol has very good performance in various pharmacological activities, including inhibition of inflammation, regulation of oxidative stress, tumor suppression and inhibition of fungal growth [4, 5], and also used for the prevention and treatment of cancer [6].

Previous studies have shown that magnolol inhibits the growth of pathogenic microorganisms, such as *Streptococcus* [7], *Candida albicans* [8], and *Cryptococcus neoformans*, but the exact mechanism of action has not been studied. The study of the antifungal mechanism of magnolol against *Cryptococcus neoformans* can provide a theoretical basis for the treatment of *Cryptococcus neoformans* infectious diseases and lay a theoretical foundation for the search of new antifungal drugs.

## 2. Materials and Methods

### 2.1. Materials

#### 2.1.1. Strains

*Cryptococcus neoformans* standard strains (*C. neoformans* BNCC225501) were studied. This study has been approved by the Ethics Committee of the First Affiliated Hospital of Dalian Medical University (Approval No. PJ-KS-KY-2021-276).

#### 2.1.2. Antifungals and Chemical Agents

Magnolol (purity ≥ 98%), fluconazole (FLZ), urea, agar, urease agar base, YPD medium, anhydrous sodium sulfate, glycerol (GC ≥ 99%), and anhydrous D-glucose were purchased from Beijing Solarbio Science & Technology Co., Ltd., China. ATB FUNGUS 3 was purchased from bioMérieux, China. Black millets were obtained from the Taobao Shan Yu Bird Paradise store, China. Roswell Park Memorial Institute (RPMI) 1640 and Dulbecco's modified Eagle's medium (DMEM) were purchased from Thermo Fisher Scientific, USA.

#### 2.1.3. Reagent Preparation

##### 2.1.3.1. Niger Seed Medium

Weigh 7 g of black millet, put it into a cleaned mortar and pestle, grind it fully until powder, pour it into a pot, add 200 mL of purified water into the pot, bring it to boil and keep it boiling for 5 min, let it stand and wait for the temperature to drop to room temperature, then filter it through eight layers of gauze, add 0.1 g of glucose in the supernatant to make it dissolve and mix it well, then add purified water to make it volume to 100 mL, then add 2 g of Agar, and autoclave at 116°C for 30 min.

### 2.2. Experimental Methods

#### 2.2.1. Culture and Preservation of *Cryptococcus neoformans*

##### 2.2.1.1. Purification Culture

Pick a single colony by inoculation ring, three-zone delineation, inoculate in Sabouraud Agar Plate medium (SDA), and place in a 35°C incubator culture for 72 h.

##### 2.2.1.2. Culture Preservation

Collect *Cryptococcus neoformans* after purification and isolation into a special fungal preservation tube with a disposable sterile inoculation ring, and freeze and store in a −80°C refrigerator for spare.

#### 2.2.2. Drug Sensitivity Test of Common Antifungal Drugs

The inhibitory effects of common antifungal drugs on *Cryptococcus neoformans* were detected by using ATB FUNGUS 3 test paper.

#### 2.2.3. Magnolol Anti-*Cryptococcus neoformans* Drug Sensitivity Test

##### 2.2.3.1. Drug Preparation

Appropriate amounts of magnolol standard and FLZ standard were fully dissolved in DMSO stock solution and then made into 2 times final concentration with RPMI1640 medium, respectively.

##### 2.2.3.2. Preparation of Fungal Suspension

Use a swab to pick the test colonies with a colony diameter greater than 1 mm on the culture medium, inoculate them into 2 mL of saline, shake for at least 15 s, and then determine the concentration of the fungal suspension using a McFarland turbidity analyzer at 590 nm wavelength to obtain an initial fungal suspension (0.5 McFarland). This procedure will yield a yeast suspension of 1 × 10^6^–5 × 10^6^ CFU/mL. Subsequently, the working suspension (1 × 10^3^–5 × 10^3^ CFU/mL) is prepared by diluting the initial suspension 1:50 with sterile saline, followed by a secondary dilution of 1:20 with RPMI-1640 medium.

##### 2.2.3.3. Culturing Method

The minimum inhibitory concentration (MIC) was determined on the basis of the broth microdilution method, according to document M27-A2 of the Clinical Laboratory Standards Institute (CLSI). Magnolol was diluted in RPMI-1640 medium at concentrations ranging from 1 to 256 μg/mL. The negative control was the medium only without *Cryptococcus neoformans*, and the positive control was the medium plus inoculum. The incubation time was 72 h at 35°C. The lowest concentration of the antifungal agent that was able to inhibit growth by 100% with respect to the positive control was considered the MIC. The MIC for FLZ was also determined according to M27-A2. The lowest concentration of the antifungal agent that was able to inhibit growth by 50% with respect to the positive control was considered the MIC.

#### 2.2.4. The Microdilution Checkerboard Method to Detect the Synergistic Ability of Magnolol With FLZ [9]

The interaction of magnolol with FLZ against fungi was studied by the microdilution checkerboard method. Serial dilutions of FLZ were inoculated horizontally, and serial dilutions of magnolol were inoculated vertically. The results were interpreted after 72 h of incubation at 35°C.

#### 2.2.5. Time-Kill Studies

To test the inhibitory effect of magnolol on *Cryptococcus neoformans*, *Cryptococcus neoformans* was cultured in RPMI 1640 medium containing different concentrations of magnolol and FLZ, the concentration was adjusted to 1 × 10^3^ CFU/mL–5 × 10^3^ CFU/mL, and the control group was the group of adding solvent of drug DMSO. Quantitative fungal solution was taken every 12 h to smear the plate and observe the number of surviving colonies.

#### 2.2.6. Urease and Melanin Assays of *Cryptococcus neoformans*

##### 2.2.6.1. Urease Assays

1. Pick a single colony of *Cryptococcus neoformans* on the purified plate and inoculate it into YPD liquid medium, incubate at 35°C for 24 h.
2. Take 1 mL in YPD medium after 24 h incubation, centrifuge for 5 min, and discard the supernatant.
3. Add 1 mL of ddH_2_O to the precipitated organisms and resuspend, centrifuge for 5 min, discard the supernatant, and repeat the process three times.
4. Dilute the resuspended *Cryptococcus neoformans* solution to 0.5 MCF using a McFarland turbidity analyzer at 590 nm wavelength, and take 100 µL drops into Urease Agar Base medium containing different concentrations of magnolol.
5. Incubate at 35°C and observe the results after 3 days.

##### 2.2.6.2. Melanin Assay

1. Pick a single colony of *Cryptococcus neoformans* on the purified plate, inoculate it into YPD liquid medium, and incubate at 35°C for 24 h.
2. Take 1 mL in YPD medium after 24 h incubation, centrifuge for 5 min, and discard the supernatant.
3. Add 1 mL of ddH_2_O to the precipitated organisms and resuspend, centrifuge for 5 min, discard the supernatant, and repeat the process three times.
4. Dilute the resuspended *Cryptococcus neoformans* solution to 0.5 MCF using a McFarland turbidity analyzer at 590 nm wavelength and take 100 µL drops into Niger Seed medium containing different concentrations of magnolol.
5. Incubate at 35°C and observe the results after 3 days.

#### 2.2.7. Metabolism and Transcriptomics Sample Preparation

1. Single colony of *Cryptococcus neoformans* standard strain BNCC225501 was subjected to activation culture for 3 days and then examined microscopically using a microscope;
2. The single colony of *Cryptococcus neoformans* standard strain after activation culture was inoculated into 100 mL of YPD culture and incubated at 35°C for 12 h;
3. Transfer the cultured YPD medium containing *Cryptococcus neoformans* into 50 mL of EP, centrifuge at 4500 rpm for 5 min to collect the fungi, and discard the supernatant;
4. Add ddH_2_O to the centrifuged fungal precipitate, resuspend, centrifuge at 4500 rpm, 5 min to collect the organisms, discard the supernatant, and repeat twice;
5. The fungal fluids were added into RPMI 1640 liquid medium containing MIC concentration of magnolol and the same volume of DMSO, so that the final concentration of the fungal fluids was 2 × 10^7^ CFU/mL, and incubated at 35°C for 2 h. There were 6 biological replicates in metabolic group, and 3 biological replicates in transcriptional group. In this study, the addition of magnolol was considered as group H, and the addition of DMSO was recognized as group C;
6. Discard the supernatant and store at −80°C.

#### 2.2.8. Metabolomic Testing and Analysis

The samples obtained in Section 2.2.7 were analyzed for metabolites using an untargeted metabolomics approach. Intersample metabolites were taken for principal component analysis (PCA) to observe data distribution and aggregation; fold change and orthogonal partial least squares-discriminant analysis (OPLS-DA) modeling of the variable importance in project (VIP) values were combined to screen differential metabolites, and pathway enrichment analysis of differential metabolites was performed using Kyoto Encyclopedia of Genes and Genomes (KEGG) database.

#### 2.2.9. Transcriptome Sequencing and Analysis

Differential expression analysis of genes was performed using differential analysis software edgeR to obtain genes with differential expression between comparative groups based on certain standardized processing and screening conditions. After obtaining the differentially expressed genes, the differential genes were analyzed for functional classification by comparing with databases to obtain the roles of these differential genes in the cells and the metabolic pathways involved. Annotated databases include Gene Ontology (GO) database and KEGG database.

#### 2.2.10. Network Pharmacology

Predicted targets for magnolol were obtained from the TCMSP database, and major target genes associated with fungi were obtained from the GeneCards database. Intersecting target genes were obtained using Venny 2.1.0 online tool (https://bioinfogp.cnb.csic.es/tools/venny/). The target protein crystal structures were then preprocessed using AutoDock Vina software (http://vina.scripps.edu/), including removal of hydrogenation, modification of amino acids, optimization of energies, and adjustment of force field parameters. Finally, the target structure was molecularly docked to the magnolol structure using the Vina software within the PyRx software, where the affinity (kcal/mol) value represents the binding capacity of the two bindings; the lower the binding capacity, the more stable the ligand–receptor binding. The 2D plots were visualized using Pymol and Discovery Studio 2020 Client.

### 2.3. Statistical Analysis

SPSS 22 software was used to analyze the data, and GraphPad Prism 9 and Fig Draw software were selected to plot the results.

## 3. Results

### 3.1. In Vitro Antifungal Activity of Antifungal Drugs

Susceptibility tests to five antifungal drugs, 5-fluorocytosine (5-FC), amphotericin B (AMB), FLZ, itraconazole (ITRA), and voriconazole (VRC), were performed on *Cryptococcus neoformans*. The results are shown in Table 1.

### 3.2. In Vitro Antifungal Activity of Magnolol Against *Cryptococcus neoformans*

The MIC of magnolol against *Cryptococcus neoformans* strain was 8 μg/mL; the microdilution checkerboard method was used to investigate magnolol interactions with FLZ against *Cryptococcus neoformans*. The results show that the magnolol and FLZ combination revealed good synergistic effects against *Cryptococcus neoformans* (FIC ≤ 0.5). The results are shown in Table 2.

### 3.3. Time–Kill Curve

**Figure 1:**
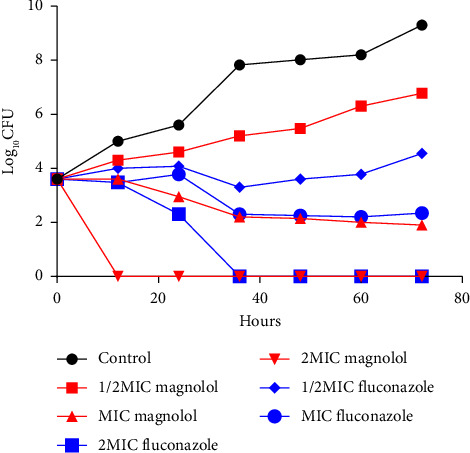
Inhibitory and fungicidal characteristics of different concentrations of magnolol and fluconazole against *Cryptococcus neoformans*.

The time–kill curve for 0–72 h demonstrated that magnolol at 0.5 MIC and 1 MIC exerted an inhibitory effect on *Cryptococcus neoformans*. However, 2 MIC exerted fungicidal effects. The fungicidal effect of magnolol was positively correlated with the concentration of magnolol, as shown in Figure 1.

### 3.4. Effect of Magnolol on the Main Virulence Factors of *Cryptococcus neoformans*

**Figure 2:**
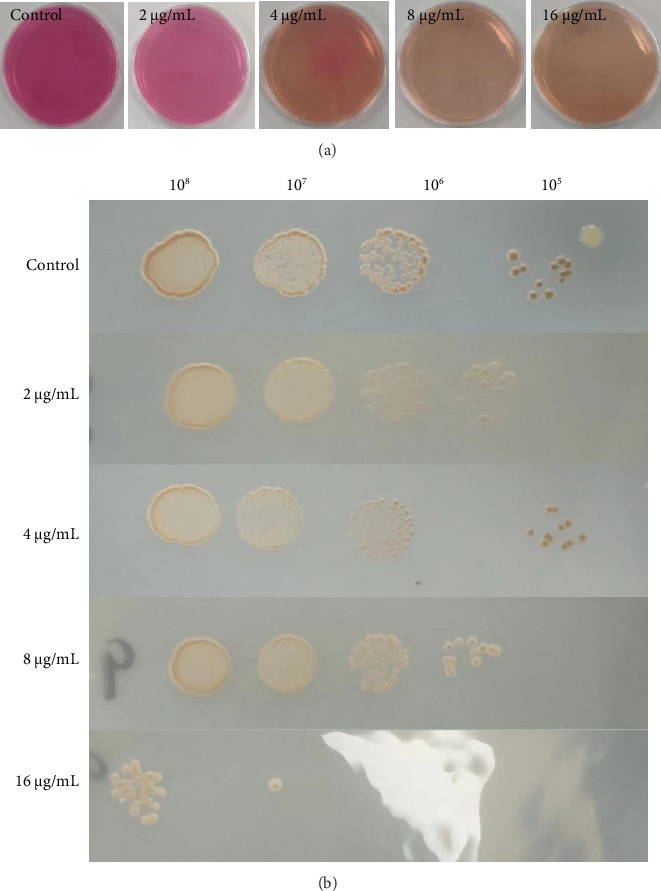
Effect of magnolol on the main virulence factors of *Cryptococcus neoformans*. (a) Expression of urease under the effect of different concentrations of magnolol and (b) expression of melanin under the effect of different concentrations of magnolol.

Urease plays an important role in the ability of *Cryptococcus neoformans* to colonize the lungs [10]and cross the blood–brain barrier [11]. In the present study, *Cryptococcus neoformans* was dropped into Urease Agar Base medium containing different concentrations of magnolol and incubated at 35°C for 72 h. The result showed that when the magnolol concentration reached 8 μg/mL, it inhibited urease expression in *Cryptococcus neoformans*. To reveal the effect of magnolol on melanin production in *Cryptococcus neoformans*, the study measured melanin production using Niger seed medium. The results showed that magnolol did not affect melanin production in *Cryptococcus neoformans*, as shown in Figure 2.

### 3.5. Results of Metabolomics Under the Effect of Magnolol

**Figure 3:**
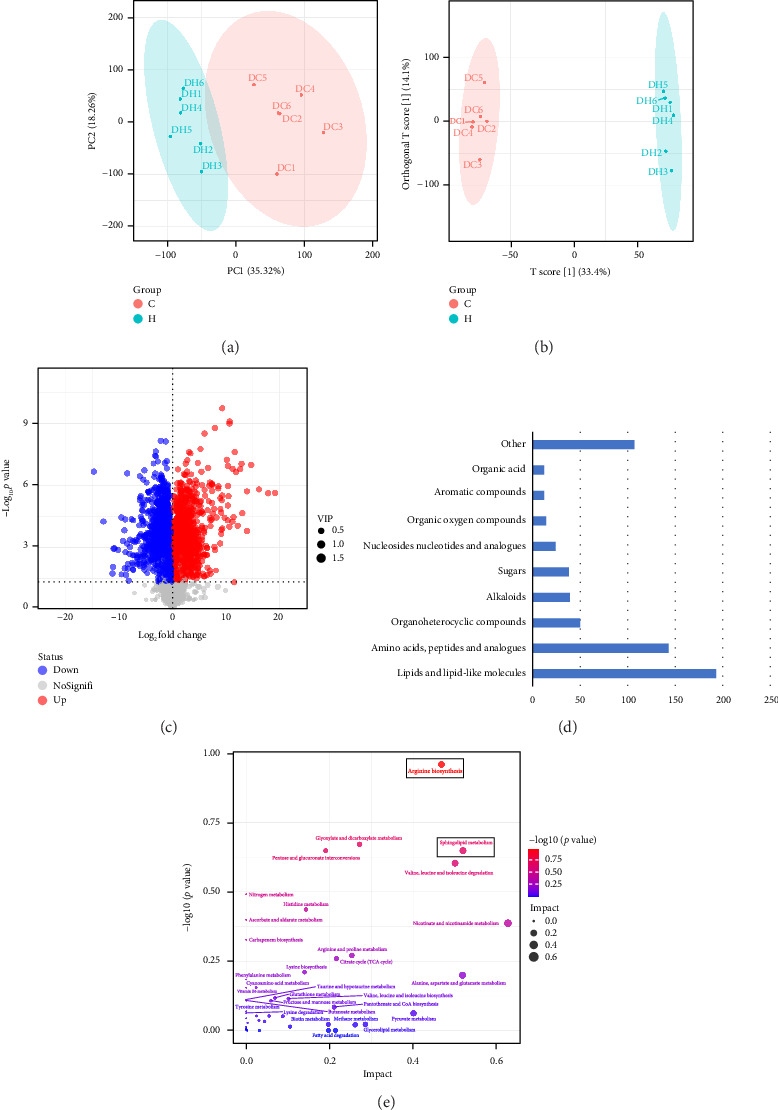
Metabolomics results. (a) Score scatter plot of PCA, (b) score scatter plot of OPLS-DA model, (c) volcano plot. Each point in the volcano plot represents a metabolite, the horizontal coordinate represents the fold change of each substance in the group comparison (taking the logarithm of the base 2), the vertical coordinate represents the *p* value of the test *p* value (negative logarithm with a base of 10), and the size of the scatter represents the VIP value of the OPLS-DA model; the larger the scatter, the larger the VIP value. Scatter color represents the final screening results, with significantly upregulated metabolites in red, significantly downregulated metabolites in blue, and nonsignificantly different metabolites in gray, (d) differential metabolite classification map, and (e) KEGG enrichment of differential metabolites. Each bubble represents a metabolic pathway. The horizontal coordinate is the magnitude of the influencing factor in topological analysis. The vertical coordinate is the *p* value of the enrichment analysis.

Liquid chromatography–mass spectrometry (LC-MS) was used to detect changes in the metabolism of *Cryptococcus neoformans* under different culture conditions. PCA showed that the two groups were clearly separated (Figure 3(a)), and OPLS-DA indicated that the model was robust (Figure 3(b)). We visualized the results of screening differential metabolites as volcano plot (Figure 3(c)). Among the differential metabolites, lipids and lipid-like molecules were found to be the highest percentage of differential metabolites, except for unclassified metabolites. A total of 30% of the metabolites belonged to lipids and lipid-like molecules, followed by amino acids, peptides, and analogues (22.63%), organoheterocyclic compounds (7.91%), alkaloids (6.17%), sugars (6.01%), nucleosides, nucleotides, and analogues (3.80%), organic oxygen compounds (2.22%), aromatic compounds (1.90%), organic acids (1.90%), and other (16.93%). As shown in Figure 3(d). KEGG pathway enrichment analysis was performed based on the differential metabolite results. The results showed that the metabolites were mainly enriched in glyoxylate and dicarboxylate metabolism, arginine biosynthesis, valine, leucine, and isoleucine degradation, arginine and proline metabolism, cysteine and methionine metabolism, pentose and glucuronate interconversions, sphingolipid metabolism, histidine metabolism, and citrate cycle (Figure 3(e)).

#### 3.5.1. Effect of Magnolol on Arginine Biosynthesis

**Figure 4:**
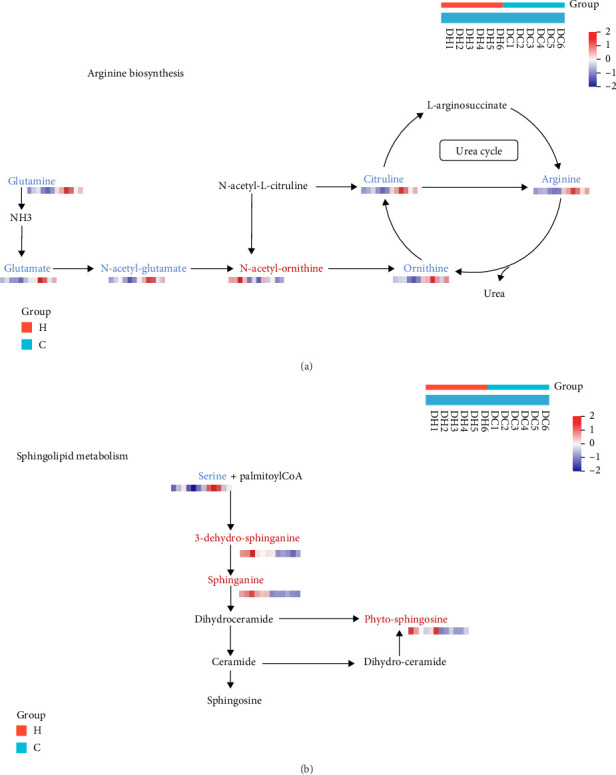
Altered metabolic pathways. (a) Arginine biosynthesis pathway and (b) sphingolipid metabolism, red: metabolites with increased levels in the *Cryptococcus neoformans* treated with magnolol, blue: metabolites with a decreased level in the *Cryptococcus neoformans* treated with magnolol, H: magnolol treatment group, C: control group.

In metabolomics, we found that the metabolites arginine, ornithine, and acetylglutamate were significantly downregulated, and we projected these metabolites into the metabolic pathway and found that they mainly affected arginine biosynthesis metabolism (Figure 4(a)), which is an important raw material for protein synthesis, an intermediate metabolite in a number of metabolic pathways, and has an important physiological and biochemical function [12]. The process of arginine synthesis in fungi can generally be divided into 3 main parts: formation of ornithine from glutamic acid by a series of enzymes, synthesis of carbamoyl phosphate, and synthesis of arginine from ornithine and carbamoyl phosphate. The metabolism of normal arginine-producing strains is maintained in a balanced steady state, and we inferred from the metabolomics results that magnolol could play an antifungal role by affecting arginine metabolism and thus strain metabolism.

#### 3.5.2. Effect of Magnolol on Sphingolipid Metabolism

In metabolomics, we found that the metabolites 3-dehydro-sphinganine, sphinganine, and phyto-sphingosine were significantly upregulated. We projected these metabolites into the metabolic pathway and found that they primarily affected sphingolipid metabolism (Figure 4(b)). Sphingolipid metabolism refers to the processes that maintain the homeostasis of sphingolipids, including their synthesis, degradation, and reuse. Cells convert galactoglycerolipids and choline glycerophosphate into sphingolipids through the catalysis of various enzymes. The degradation of sphingomyelin is catalyzed by sphingomyelinases, which break down sphingomyelin into metabolites such as phosphocholine, ceramides, and hemolysins. Sphingolipid reuse means that metabolites produced during sphingomyelin metabolism can be resynthesized into sphingomyelin. Sphingolipids have many other roles as structural lipids of cell membranes, acting as a source of ceramides in cell signaling and apoptosis [13, 14], and forming clusters or nanostructural domains with cholesterol and ceramides. The metabolic status of sphingolipids plays an important role in maintaining the stability of cell membranes and normal cell signaling processes [15]. The increase in sphingolipid metabolites may be due to cell membrane damage. From the metabolic results, we deduced that magnolol could have an antifungal effect by affecting sphingolipid metabolism and, consequently, the metabolism of the strain.

### 3.6. Results of Transcriptomics Under the Effect of Magnolol

**Figure 5:**
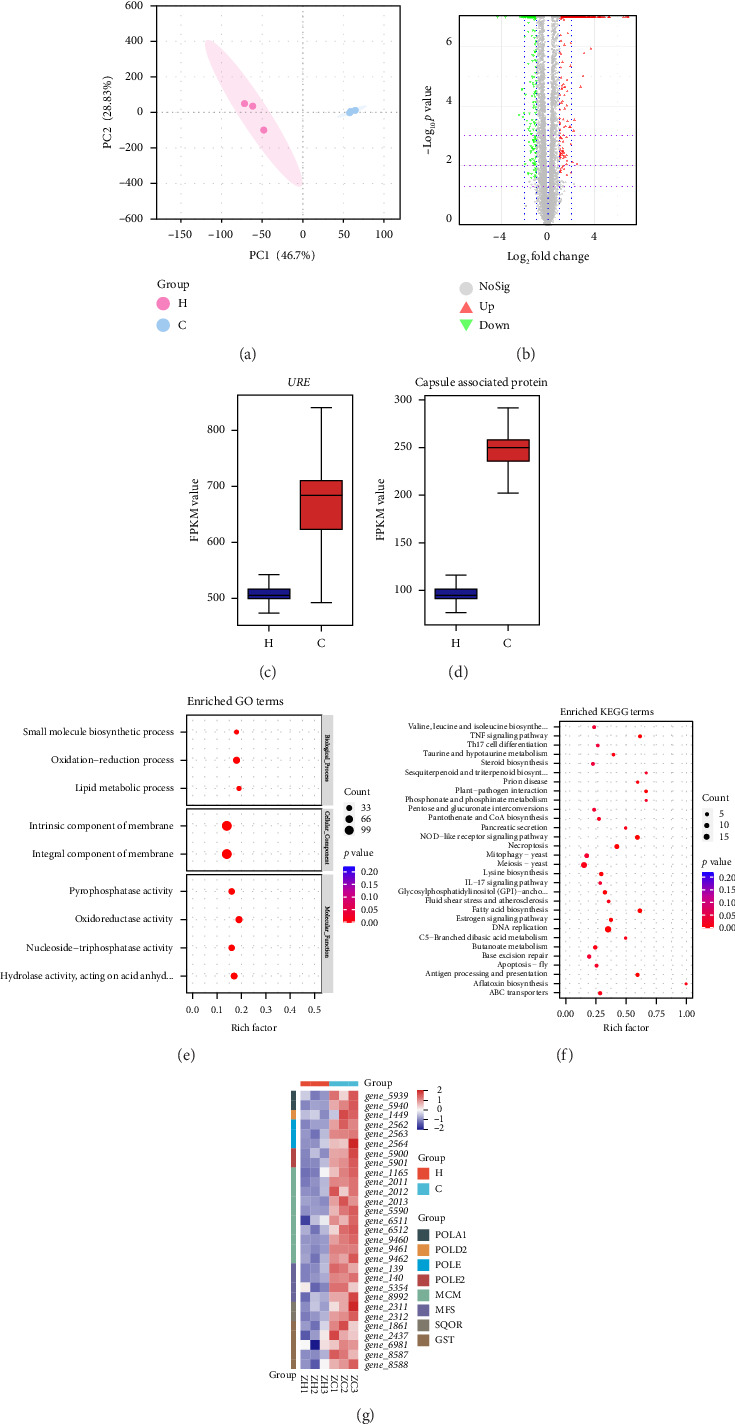
Transcriptomics results. (a) PCA map, (b) differential gene volcano map, (c) differential expression of URE in comparisons H:C, (d) differential expression of capsule-associated protein gene in comparisons H:C, (e) GO functional enrichment analysis statistics map, (f) KEGG functional enrichment analysis statistic map, and (g) differential gene heatmap, H: magnolol treatment group, C: control group.

The results showed that PCA revealed good intragroup replication (Figure 5(a)), and there was significant variability among different groups in the PCA. We used differential expression analysis software edgeR to perform differential expression analysis of genes, and screening (FDR ≤ 0.05 and |log2FC| ≥ 1) revealed that a total of 928 genes were detected to be altered under the effect of magnolol (Figure 5(b)). After the effect of magnolol, we found that the *Cryptococcus neoformans* urease–producing capacity decreased, which was also demonstrated in transcriptomics (Figure 5(c)). In the phenotyping experiments, the culture conditions may have been insufficient. The capsule of *Cryptococcus neoformans* was not successfully induced. However, a significant reduction in the transcription of capsule-associated proteins was observed in the transcriptomic analysis (Figure 5(d)). The results were analyzed by GO enrichment analysis, which showed that the gene enrichment in the magnolol group compared to the blank group was mainly related to oxidation–reduction process and lipid metabolic process at the level of biological process; at the level of cellular component, it was mainly related to integral component of membrane and intrinsic component of membrane; and at the level of molecular function, it was mainly related to oxidoreductase activity (Figure 5(e)).

KEGG enrichment analysis showed that the differentially genes were mainly enriched in the DNA replication, NOD-like receptor signaling pathway, and fatty acid biosynthesis (Figure 5(f)).

#### 3.6.1. Effect of Magnolol on DNA Replication

Magnolol affects *Cryptococcus neoformans* interfering with DNA replication possibly by affecting the synthesis of minichromosome maintenance proteins (MCMs) and DNA polymerase (Figure 5(g)). Transcriptomics results showed that the expression of minichromosome maintenance protein 2 (MCM2), minichromosome maintenance protein 3 (MCM3), minichromosome maintenance protein 4 (MCM4), minichromosome maintenance protein 6 (MCM6), minichromosome maintenance protein 7 (MCM7), DNA polymerase alpha subunit A (POLA1), DNA polymerase delta subunit 2 (POLD2), DNA polymerase epsilon catalytic subunit A (POLE), and DNA polymerase epsilon subunit B (POLE2) were significantly downregulated.

The MCM family is a group of ubiquitous and highly conserved proteins first identified in yeast minichromosome deletion mutants. Their functions are mainly related to the initiation of DNA replication [16]. The six proteins (MCM2–7) are involved in the formation of the preinitiation complex (PIC) of replication. MCM4/6/7 has helicase activity and DNA-dependent ATPase activity, which is the catalytic core of MCMs complexes. There is now growing evidence that MCMs play a very important role not only in DNA replication extension but also in transcription, chromosome remodeling, and genome stability. DNA polymerase is an enzyme that plays an important role in cellular replication of DNA and is key to maintaining the stability of an organism's genome [17–19]. It mainly uses DNA as a template for replication, synthesizing new DNA from the 5′ end to the 3′ end of the template strand. DNA polymerase epsilon is one of the major DNA polymerases in eukaryotic nuclear genomes. It is mainly responsible for eukaryotic genome replication. When DNA replication is under stress, DNA synthesis stops until the stress is ameliorated [19]. DNA polymerase epsilon is involved in DNA repair. Recent studies in yeast, using whole-genome sequencing and other methods, have shown that reduced levels of DNA polymerase epsilon in yeast greatly increase the incidence of mitotic recombination, aneuploidy, and single-base mutations, leading to genomic instability.

#### 3.6.2. Effect of Magnolol on Transmembrane Transport

Magnolol affects *Cryptococcus neoformans* by interfering with transmembrane transport possibly by affecting the synthesis of The Major Facilitator Superfamily (MFS). Transcriptomics results showed that the expression of MFS was significantly downregulated (Figure 5(g)).

MFS is one of the largest families of membrane transporter proteins, and MFS transporter proteins are widely distributed throughout the biological world [20]. Their functions have been associated with numerous life activity phenomena. The primary function of MFS transporter proteins is to facilitate the transmembrane transport of substances and to participate in a series of conformational changes during substrate transport [21]. These proteins can selectively transport a wide range of substrates across biological membranes and are capable of utilizing the energy stored in transmembrane electrochemical gradients to transport a broad spectrum of substrates with different physicochemical properties. Additionally, they play key roles in a variety of physiological processes [22].

#### 3.6.3. Effect of Magnolol on Oxidation–Reduction Process

Magnolol affects *Cryptococcus neoformans* may interfere with oxidation–reduction process by affecting the synthesis of glutathione transferase (GST) and sulfide:quinone oxidoreductase (SQOR). Transcriptomics results showed that the expressions of GST and SQOR were significantly downregulated (Figure 5(g)).

GST is an enzymatic antioxidant found in almost all organisms. GST is involved in the synthesis and metabolism of a wide range of endogenous substances [23]. GST catalyzes the binding of glutathione to many exogenous compounds for detoxification [24]. SQOR is a member of the GSH reductase superfamily, a key enzyme in the oxidative detoxification of sulfide. H_2_S is a product of microbial metabolism, and higher levels of H_2_S inhibit complex IV in the electron transport chain [25]. SQOR prevents H_2_S accumulation and generates highly reactive persulfide species as products, which can be further oxidized or can modify cysteine residues in proteins via persulfidation.

### 3.7. The Results of Combined Metabolomics and Transcriptomics

**Figure 6:**
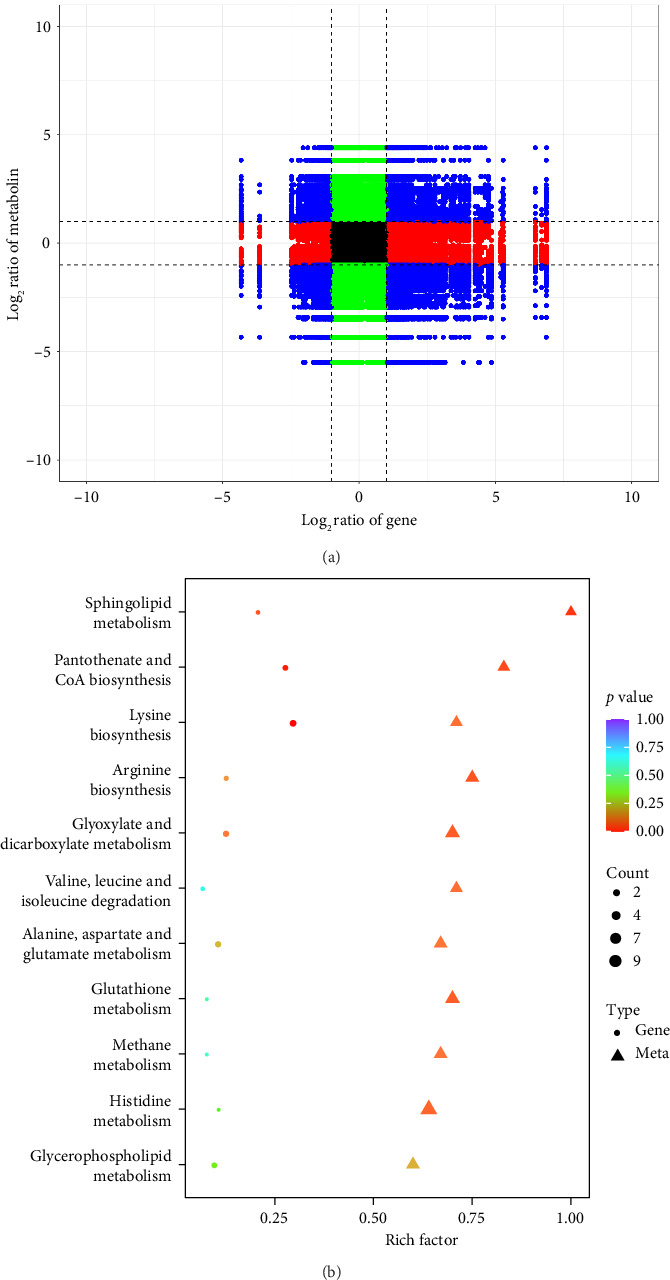
Results of the combined analysis. (a) The genes correlation and components between H and C are shown by the nine-quadrant diagram and (b) KEGG enrichment analysis of DEGs and DAMs enriched in the same pathway.

The transcriptome is an important method for obtaining gene expression in organisms, and the metabolome is the basis and direct embodiment of an organism's phenotype. Correlating gene expression and metabolic phenotype for joint analysis can help rapidly target key genes and metabolic pathways. The metabolome and transcriptome correlation analysis enables the mutual validation of genomes, which not only predicts metabolite changes at the transcriptional level but also verifies the results of gene transcription at the metabolic level and explores the causal relationship between genes and metabolites. By combining functional annotation and metabolic pathway enrichment with biofunctional analyses, the causal relationship between genes and metabolites can be explored, and key metabolic pathways can be targeted. Additionally, integrating functional annotation and metabolic pathway enrichment allows us to identify key metabolic pathways, genes, and metabolites and systematically analyze the relationship between biological regulatory mechanisms and biomolecular functions. To deeply investigate the mechanism of magnolol against *Cryptococcus neoformans*, the experiment was analyzed by combining data from transcriptomics and metabolomics, DEGs and DAMs were correlated (Figure 6(a)), and pairwise comparisons of enrichment pathways were performed (Figure 6(b)). The analysis revealed that GPL metabolism and glutathione metabolism were significantly altered following magnolol treatment.

#### 3.7.1. Combined Metabolomics and Transcriptomics Analysis of the Effects of Magnolol on GPL Metabolic Pathways

**Figure 7:**
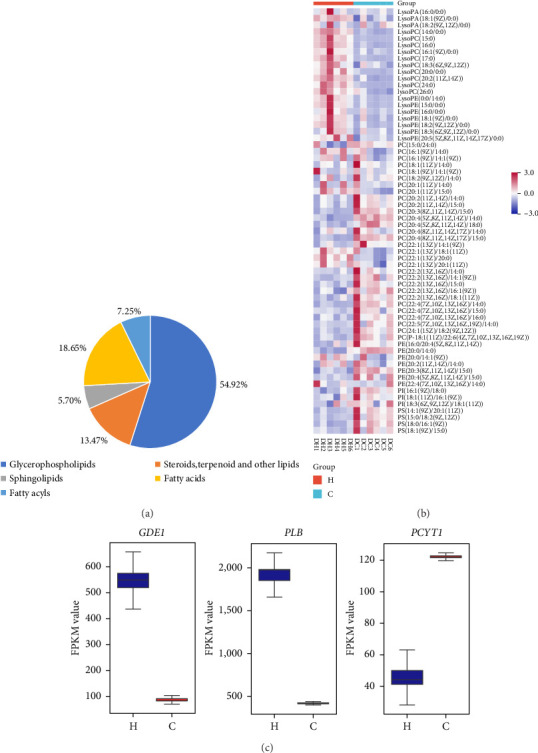
Differential metabolites and genes of glycerophospholipid metabolic pathway under the effect of magnolol. (a) Lipids and lipid-like molecules metabolite classification map, (b) differential metabolites of glycerophospholipids under the effect of magnolol, and (c) differential expression of *GDE1, PLB*, and *PCY1* in comparisons H:C, H: magnolol treatment group, C: control group.

Metabolomics analysis revealed that magnolol had a significant impact on lipids and lipid-like molecules, with GPLs (54.92%), fatty acids (18.65%), steroids, terpenoids, and other lipids (13.47%), fatty acyls (7.25%), and sphingolipids (5.70%). GPLs were the most affected metabolites in the molecular class of lipids and lipid-like molecules (Figure 7(a)). Phospholipids, which are complex lipids commonly found on the surface of cell membranes, are primarily characterized by their role in forming lipid bilayers along with proteins, glycolipids, and cholesterol. Phospholipids can be divided into two major groups: GPLs and sphingolipids, which are composed of glycerol and sphingol, respectively. GPLs include phosphatidylethanolamine (PE), phosphatidylinositol (PI), phosphatidylserine (PS), phosphatidylglycerol (PG), and phosphatidylcholine (PC). Lysophospholipids (LPLs) are a class of lipid metabolism intermediates in cell membranes, primarily generated through the hydrolysis of phospholipid molecules. Common LPLs include lysophosphatidic acid (LysoPA), lysophosphatidylcholine (LysoPC), lysophosphatidylethanolamine (LysoPE), lysophosphatidylglycerol (LysoPG), lysophosphatidylserine (LysoPS), and lysophosphatidylinositol (LysoPI). The data from this study indicated that magnolol significantly affected the metabolism of GPLs, leading to a downregulation of GPL content and a general increase in LPL content (Figure 7(b)). Transcriptomic analysis revealed that enzymes associated with GPL metabolism (Figure 7(c)) were also altered.

#### 3.7.2. Combined Metabolomics and Transcriptomics Analysis of the Effects of Magnolol on Glutathione Metabolic Pathways

**Figure 8:**
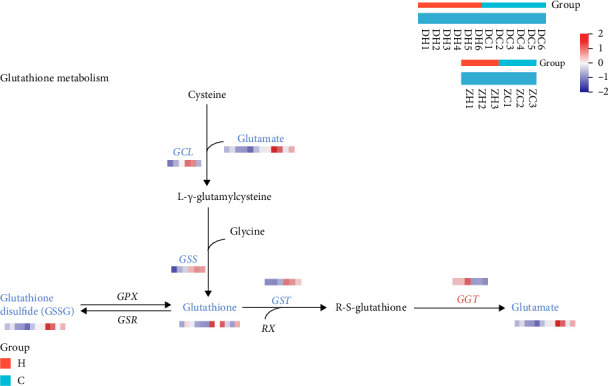
Differential metabolites and genes of glutathione metabolic pathway under the effect of magnolol, red: metabolites/genes with increased levels in the *Cryptococcus neoformans* treated with magnolol, blue: metabolites/genes with a decreased level in the *Cryptococcus neoformans* treated with magnolol, H: magnolol treatment group, C: control group.

Data analysis showed significant alterations in genes related to redox processes in the transcriptomics, among which glutathione metabolism caught our attention. Combined metabolomics and transcriptomics analysis revealed significant alterations in the glutathione metabolism pathway (Figure 8).

Glutathione (GSH) is a major nonenzymatic compound that protects cells from oxidative stress. GST catalyzes the binding of glutathione to many exogenous compounds for detoxification.

Analysis of the metabolomics data revealed that the levels of the metabolites glutathione, glutathione disulfide, L-glutamine, and L-glutamic acid were significantly downregulated after magnolol action. Glutamine can be converted to glutamic acid by glutaminase; L-glutamine and L-glutamic acid are precursors for the synthesis of GSH, a tripeptide composed of L-glutamic acid, cysteine, and glycine. Glutathione exists in both reduced and oxidized forms, and glutathione peroxidase (GPX) and glutathione reductase (GSR) catalyze the interconversion between these two forms [23, 26], downregulation of GSH synthase (GSS), GST, and glutamate-cysteine ligase catalytic subunits (GCLC) expression was found in the analysis of transcriptomics data.

GSH synthesis proceeds through a two-step, ATP-dependent enzymatic process. The first step is catalyzed by glutamate-cysteine ligase (GCL), which consists of GCLC and GCLM. GCL combines cysteine with glutamate to produce γ-glutamylcysteine (γ-GC). The second step is catalyzed by GSS, which adds glycine to γ-glutamylcysteine to form GSH. GSH exerts negative feedback inhibition of GCL. After coenrichment analysis, it was found that the glutathione metabolism pathway was affected, so we inferred that the content and metabolism of GSH were affected by the action of magnolol.

### 3.8. Network Pharmacology Explores the Targets of Antifungal Effect of Magnolol

**Figure 9:**
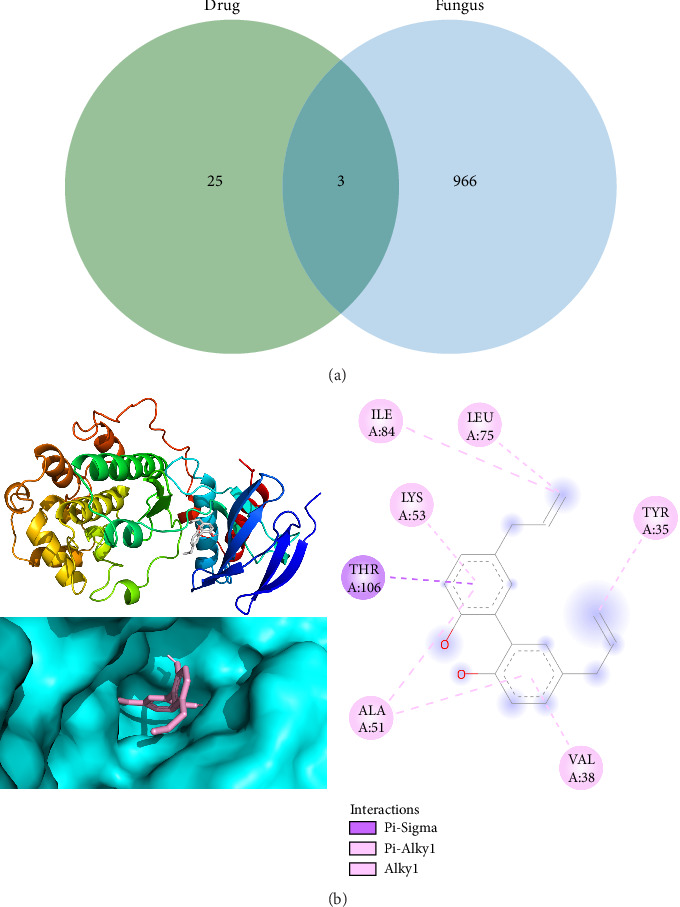
Network pharmacology results. (a) Drug–fungal cross-talk target genes and (b) docking results of magnolol and MAPK14.

Magnolol predicted targets were obtained from the TCMSP database (Table 3), and a total of 969 fungal genes were found in the GeneCards database using the keyword “fungi”. The 31 magnolol target genes and 969 fungal genes, extracted from the TCMSP database, were imported into the Venny 2.1.0 online tool (https://bioinfogp.cnb.csic.es/tools/venny/), and three intersecting target genes, DPP4, MAPK14, and CCNA2, were obtained (Figure 9(a)). These genes are potential targets for magnolol to inhibit fungal infections. By reviewing the literature, this study found that previous research has shown that the antifungal activity of magnolol is associated with the MAPK pathway. Therefore, in this study, MAPK14 was selected for molecular docking, and then using AutoDock Vina software (http://vina.scripps.edu/), the crystal structure of MAPK14 was preprocessed, including removal of hydrogenation and modification of amino acids, optimization of energy, and adjustment of force field parameters. Finally, this target structure was molecularly docked to the magnolol structure using Vina within the PyRx software. The affinity (kcal/mol) value represents the binding capacity of the two bindings; the lower the binding capacity, the more stable the ligand-receptor binding. Pymol was used to visualize the analysis, and the 2D plots were visualized using Discovery Studio 2020 Client.

As shown in Figure 9(b), magnolol forms a Pi-Sigma interaction with THR106 of MAPK14 and hydrophobic interactions with ALA51, CYS251, ILE84, VAL38, TYR35, LEU75, and LYS53. Docking results showed a docking binding energy of −7.3 kcal/mol for MAPK14 and magnolol.

## 4. Discussion–Parei Aqui

In recent years, the morbidity and mortality rates of invasive fungal infections have remained high. This is because there are relatively few drugs available for the treatment of infections, and these drugs are expensive, with relatively high side effects and often drug–drug interactions [27]. It has become a current research hotspot to extract new antifungal drugs from Chinese herbs with low side effects, low price, good efficacy, and not easy to be drug-resistant.

Magnolol is an effective antifungal component and one of the major active compounds of *Magnolia officinalis* Rehd. et Wils. It has been reported to possess biological functions such as anti-inflammatory, antifungal, anticancer [28, 29], and antioxidant activity [6, 30]. Previous studies have shown that magnolol significantly inhibits the growth of *Candida albicans*, *Staphylococcus aureus*, *Streptococcus*, and *Mycobacteria* [4, 7, 8]. Plant sources of magnolol have been widely researched due to their nontoxicity, ease of biodegradation, and environmentally safe properties.

In this study, magnolol was found to have an anti-*Cryptococcus neoformans* MIC of 8 μg/mL, and the results of the combination drug sensitivity experiments showed that the combination drug was more effective than the inhibition effect of the drug alone. Magnolol effectively inhibited urease synthesis in *Cryptococcus neoformans*, but had no significant effect on melanin production. Based on these findings, this study was conducted to further investigate the antifungal mechanism of Magnolol against *Cryptococcus neoformans* by metabolomic and transcriptomic analyses.

Transcriptomics results showed that a total of 928 genes were detected to be altered after magnolol action, with downregulation of transcription of capsular-associated proteins and urease. Enrichment analysis showed that the differential genes were enriched in cellular membrane composition and function and redox processes. Metabolomics results showed that lipids and lipid-like molecules accounted for the largest proportion of differential metabolites (30%), with GPLs having the highest proportion in lipids and lipid-like molecules.

To further investigate the antifungal mechanism of magnolol, this study conducted a combined metabolomics and transcriptomics analysis. After the combined analysis, this study found that GPL metabolism and GSH metabolism were significantly altered.

GPLs showed a broad downregulation of abundance and a significant increase in LPL after magnolol action. Phospholipids are the main components that make up cell membranes and often form phospholipid bilayers together with other molecules such as proteins, glycolipids, and cholesterol [5, 31]. It has been shown that altering phospholipid biosynthesis can show effective anti-*Cryptococcus neoformans* activity [32, 33]. The cell membrane is a barrier for the passage of hydrophilic molecules.

LPLs are usually minor components of cell membranes, and their content can be significantly elevated under environmental stress conditions, mainly generated by the hydrolysis of phospholipid molecules. It has been shown that elevated levels of LPLs in cell membranes can disrupt the membrane homeostasis and cause serious damage to the membrane structure, and the accumulation of LPLs in the cell membrane can also disrupt the lipid symmetry of the membrane, resulting in inhibition of cell growth, leakage of interstitial space, and, in extreme cases, lysis of the cell.

Studies in the transcriptome revealed significant changes in the transcription of phospholipase B (PLB), choline-phosphate cytidylyltransferase (PCYT1), and glycerophosphoryldiester phosphodiesterase (GDE1) genes. PLB belongs to the phospholipase family, which is widespread in nature and active in many microbial, protozoan, and mammalian cells. Phospholipase hydrolyzes GPLs to generate LPLs. Based on the site of hydrolysis, phospholipases are classified into four types of activity, termed phospholipase A1, A2, C, and D. PLB has both A1 and A2 activity. Evidence suggests that PLB is an important component of the virulence pool of pathogenic fungi, and plays an important role in fungal pathogenicity by degrading phospholipids in cell membranes, damaging cell membranes and thereby reducing cellular defenses and inducing inflammatory responses, and it has been found that PLB can increase the survival and proliferation rates of *Cryptococcus neoformans* in the neurological central system, as well as enhance its virulence [34].

GDE1, generally found in prokaryotic and eukaryotic organisms, is a metabolic enzyme that catalyzes the hydrolysis of glycerophosphoric acid diesters to produce small molecules such as glycerol-3-phosphate and the corresponding alcohols (e.g., choline, myo-inositol, and serine). It is a key enzyme in the phospholipid metabolism pathway that maintains G-3-P concentrations, for phospholipid remodeling and synthesis. PC is synthesized primarily through the glycerol diester pathway. PCYT1 is the key enzyme, which catalyzes the condensation of phosphorylcholine with CTP to synthesize CDP-choline. CDP-choline supplies phosphorylcholine to the diglycerides to synthesize PC. *Cryptococcus* cell membranes have sites for multiple proteins associated with fungal virulence and are involved in the production of extracellular vesicles with a potential role in virulence [35]. Therefore, magnolol may affect cell membrane stability by influencing genes involved in GPL metabolism (*PLB*, *GDE1*, and *PCYT1*).

Magnolol has a potent antifungal effect, which may be related to the inhibition of the GSH system and disruption of the redox environment in the fungus. GSH is a tripeptide containing sulfhydryl groups, formed by the combination of glutamic acid, cysteine, and glycine, and it has antioxidant and detoxifying effects.

The results showed that the GSH metabolic pathway was altered after magnolol action, and the metabolites and genes associated with the GSH metabolic pathway were significantly affected. GSH is abundantly distributed in plant, animal, and fungal cells [36] and is involved in a wide range of cellular metabolic activities [37]. GSH directly scavenges ROS, nitric oxides, and carbon free radicals. In addition, GSH can be involved in the synthesis of ergothioneine, another antioxidant catalyzed by the enzyme EgtB [37]. A significant decrease in ergothioneine was observed in the metabolomics analysis. Previous studies have shown that deletion of the *GSS* gene leads to a significant reduction in intracellular GSH levels. This also resulted in the loss of major virulence factors, including capsule and melanin production, growth at host body temperature, and increased susceptibility to antifungal drugs. The decrease in GSH content, leading to significant alterations in oxidative stress, may be the reason for magnolol antifungal [38].

GST is an enzymatic antioxidant found in almost all organisms. GST can be involved in many physiological and metabolic processes [39], such as the regulation of redox state, detoxification of metabolites, and maintenance of immune homeostasis [40]. GST is also currently being extensively studied as a potential biomarker of oxidative stress [41, 42]. Specifically, GST can catalyze the reaction between a series of exogenous and endogenous compounds and GSH, converting them into more stable metabolites. This promotes the metabolism and clearance of toxic substances and maintains the homeostasis of the intracellular environment and normal physiological functions.

**Figure 10:**
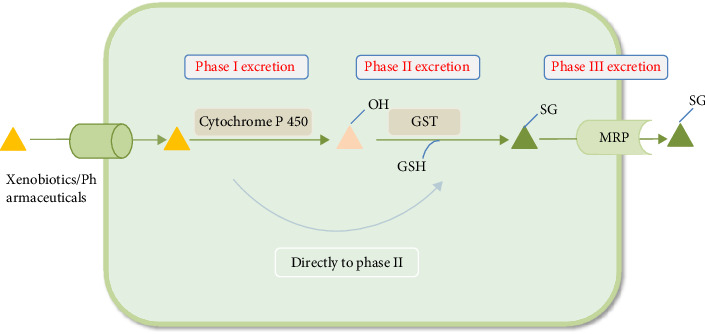
The process of xenobiotics detoxification.

GST is a Phase II detoxification enzyme in the cellular detoxification process. It plays a role in cytoprotection by catalyzing the binding of GSH to oxidative stress products such as ROS, metabolite byproducts, and xenobiotics [23], making them less toxic and facilitating their elimination from the cell. GST plays a crucial role in maintaining cellular integrity and protecting against DNA damage [43]. When xenobiotics enter the cell, the detoxification process in the cell is mainly lipophilic molecules metabolized by Phase I enzymes (cytochrome P450). The activated xenobiotics bind to GSH via the Phase II detoxification enzyme GST and are eventually exported out of the cell in Phase III via transmembrane multidrug resistance–associated proteins (MRPs) of the ABC transporter protein C family [44], and some compounds (which are polar or hydrophilic in nature) may be directly metabolized in Phase II. The detoxification process is shown in Figure 10.

Previous studies have shown that magnolol antifungal may be associated with PKC and MAPK pathways [45], which increase cell membrane permeability [46]. Magnolol affects the transition of *Candida albicans* from yeast to mycelium and biofilm formation. Previous studies have shown that magnolol disrupts redox homeostasis thereby acting as an antimicrobial agent. This study demonstrated that disruption of GSH-dependent redox homeostasis and inhibition of detoxification processes by magnolol may serve as a novel antifungal strategy. The results of this paper demonstrated the effect of magnolol on GPL and glutathione metabolic pathways. One possible explanation is that magnolol affects the glutathione metabolic pathway, and the depletion of GSH leads to the accumulation of ROS and toxic substances, causing cell membrane damage and ultimately leading to the death of the fungus. However, the manner in which magnolol affects glutathione metabolism, especially its upstream targets, needs to be further investigated.

## 5. Conclusion

**Figure 11:**
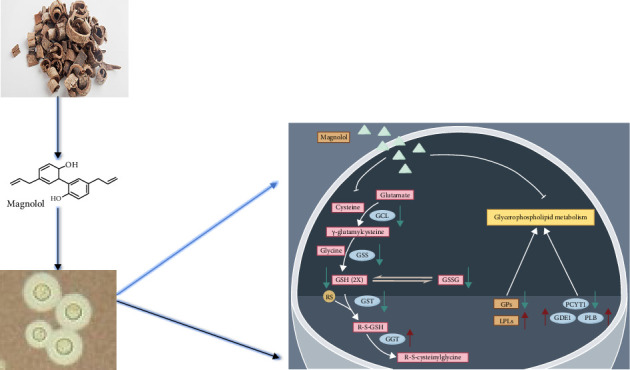
The effect of magnolol against *Cryptococcus neoformans*.

In this paper, magnolol was found to inhibit the growth of *Cryptococcus neoformans*, revealing that magnolol disrupts the ability of *Cryptococcus neoformans* urease formation. The mechanism of magnolol against *Cryptococcus neoformans* was systematically investigated using metabolomic and transcriptomic analyses. Significant alterations in the metabolic pathways of GPLs and glutathione were observed (Figure 11). The results of this study demonstrate for the first time that the antifungal mechanism of magnolol may be through affecting glutathione metabolism thereby disrupting the redox homeostasis and inhibiting the detoxification response of the fungus, Meanwhile, network pharmacology and molecular docking methods were used to predict the potential mechanism of magnolol's antifungal activity in the body, and the results indicated that the MAPK14 target is a potential target of magnolol for fungal inhibition. This study provides a theoretical basis for the further development of magnolol as a therapeutic agent against fungal infections.

## Figures and Tables

**Table 1:** In vitro antifungal activity of antifungal drugs.

Strains	MIC (μg/mL)				
	5FC	AMB	FCA	ITR	VRC
Standard strain of *C. neoformans*	< 4	< 0.5	4	< 0.25	< 0.25

*Note:* Determination criteria 5-FC (S: ≤ 4 μg/mL, I: 8–16 μg/mL, R: ≥ 32 μg/mL), AMB (S: ≤ 1 μg/mL, I: 2 μg/mL, R: ≥ 4 μg/mL), FLZ (S: ≤ 8 μg/mL, I: -, R: ≥ 64 μg/mL), ITRA (S: ≤ 0.12 μg/mL, I: -, R: ≥ 1 μg/mL), and VRC (S: ≤ 2 μg/mL, I: -, R: ≥ 4 μg/mL).

**Table 2:** In vitro antifungal activity of magnolol against *Cryptococcus neoformans*.

Strains	MIC (μg/mL) Magnolol fluconazole		FIC (μg/mL) Magnolol fluconazole		FICI
Standard strain of *C. neoformans*	8	4	1	1	≤ 0.5

**Table 3:** Predicted targets of magnolol drugs.

Molecule name	Target name	Gene name
Magnolol	Prostaglandin G/H synthase 1	PTGS1, COX1
Magnolol	Muscarinic acetylcholine receptor M3	CHRM3
Magnolol	Thrombin	—
Magnolol	Muscarinic acetylcholine receptor M1	CHRM1
Magnolol	Estrogen receptor	—
Magnolol	Androgen receptor	Ar, Nr3c4
Magnolol	Sodium channel protein type 5 subunit alpha	SCN5A
Magnolol	Peroxisome proliferator activated receptor gamma	PPARG, NR1C3
Magnolol	Prostaglandin G/H synthase 2	PTGS2,COX2
Magnolol	Retinoic acid receptor RXR-alpha	RXRA,NR2B1
Magnolol	CGMP-inhibited 3′,5′-cyclic phosphodiesterase A	PDE3A
Magnolol	5-hydroxytryptamine 2A receptor	HTR2A, 5-HTR2A
Magnolol	Sodium-dependent noradrenaline transporter	SLC6A2
Magnolol	Alpha-1A adrenergic receptor	ADRA1A, ADRA1C
Magnolol	Sodium-dependent dopamine transporter	DAT
Magnolol	Beta-2 adrenergic receptor	ADRB2
Magnolol	Sodium-dependent serotonin transporter	SLC6A4
Magnolol	Estrogen receptor beta	ESR2
Magnolol	Gamma-aminobutyric acid receptor subunit alpha-1	GABRA1
Magnolol	Dipeptidyl peptidase IV	DPP4
Magnolol	Mitogen-activated protein kinase 14	MAPK14
Magnolol	Glycogen synthase kinase-3 beta	GSK3B
Magnolol	Heat shock protein HSP 90	Hsp83
Magnolol	Cell division protein kinase 2	CDK2
Magnolol	Amine oxidase [flavin-containing] B	MAOB
Magnolol	Serine/threonine-protein kinase Chk1	Chek1
Magnolol	mRNA of PKA catalytic subunit C-alpha	—
Magnolol	Ig gamma-1 chain C region	IGHG1
Magnolol	Trypsin-1	TRYP1
Magnolol	Proto-oncogene serine/threonine-protein kinase Pim-1	PIM1
Magnolol	Cyclin-A2	CCNA2

## Data Availability

The data used to support the findings of this study are included within the article.
